# IFN-γ-Driven macrophage responses in the immunity to *Mycobacterium tuberculosis* and *Mycobacterium leprae*

**DOI:** 10.3389/fcimb.2025.1679691

**Published:** 2025-11-11

**Authors:** Mohd Imran, Ahmed S. Alshrari, Abida Khan, Abdullah R. Alzahrani

**Affiliations:** 1 Department of Pharmaceutical Chemistry, College of Pharmacy, Northern Border University, Rafha, Saudi Arabia; 2 King Salman Center for Disability Research, Riyadh, Saudi Arabia; 3 Center For Health Research, Northern Border University, Arar, Saudi Arabia; 4 Department of Medical Laboratory Technology, College of Applied Medical Sciences, Northern Border University, Arar, Saudi Arabia; 5 Department of Pharmacology and Toxicology, Faculty of Medicine, Umm Al-Qura University, Al-Abidiyah, Makkah, Saudi Arabia

**Keywords:** interferon-gamma, *Mycobacterium tuberculosis*, *Mycobacterium leprae*, T-helper type 1 cells, CD8+ T cells

## Abstract

Interferon-gamma (IFN-γ) is a key stimulator of macrophage defense against *Mycobacterium tuberculosis* (Mtb) and *Mycobacterium leprae (M. leprae)*. Both pathogens adopt measures to circumvent the effects of the immune system, weakening the impact of IFN-γ and enabling them to survive in the cells. This review synthesizes how IFN-γ overdose transacts the JAK/STAT1-IRF1-transmitter to encourage maturation of phagolysosomes, reactive oxygen and nitrogen product generation, LC3-associated phagocytosis (LAP), autophagy, and improved antigen presentation and juxtaposes these pathways in tuberculosis and leprosy. We also explain the mechanisms by which mycobacteria counter this axis, including receptor downregulation, induction of IL-10/SOCS, type I INF antagonism, and the impact of miRNA. Additionally, we assessed the translational application, emphasizing its benefits, potential risks, and sources of variability. Additionally, we discuss biomarker strategies related to IFN-γ activity, such as gene signatures associated with HIF-1 and active IFN-γ measurements, which could aid in selecting patients and tracking their treatment progress. The results show that macrophage-related processes are important for the treatment and diagnosis of TB and leprosy when they occur simultaneously. This highlights the need for safe and effective treatments that focus on the host and balance the protective and harmful effects of IFN-γ.

## 1 Introduction

Tuberculosis (TB), caused by *Mycobacterium tuberculosis* (*Mtb*), remains a major global health emergency and ranks among the top infectious disease killers worldwide. In contrast, leprosy, caused by *Mycobacterium leprae* (*M. leprae*), is no longer a global emergency but persists as a significantly neglected tropical disease in endemic regions (e.g., South Asia, Africa, and South America), with >200,000 new cases annually reported by the World Health Organization. Mtb is transmitted via inhaled droplets and establishes infection in alveolar macrophages. In immunocompetent hosts, the infection is usually contained (latent TB), whereas compromised immunity (e.g., HIV co-infection, malnutrition, and immunosuppression) permits dissemination to the lymph nodes, bone, and central nervous system (extrapulmonary TB) (Pai and Behr, 2016). In immunocompetent individuals, the infection is usually contained within the lungs and remains asymptomatic (Parrish et al., 1998). In contrast, individuals with compromised immunity, such as those with HIV co-infection, malnutrition, or those receiving immunosuppressive therapy, are more susceptible to bacterial dissemination beyond the lungs to sites such as the lymph nodes, bones, and central nervous system, leading to extrapulmonary tuberculosis (Coppola et al., 2018). Granulomatous immune containment underlies latent TB; however, approximately 5–10% of cases progress to active disease, especially in the presence of HIV co-infection, malnutrition, or iatrogenic immunosuppression (Chandra et al., 2022). Leprosy transmission is linked to prolonged close contact with untreated multibacillary patients. *M. leprae* primarily affects the skin and peripheral nerves, causing sensory-motor neuropathy and disability (Rashidi et al., 2025).

Pathogenic mycobacteria reprogram macrophage signaling (e.g., IFN-γR down-modulation, SOCS/IL-10 induction, and type-I IFN antagonism) to blunt bactericidal programs (Leseigneur et al., 2020). Research has shown that Mycobacteria use microbe-induced activation of macrophages and T-cells for survival, thus validating immunotherapy strategies, especially those targeting IFN-γ. This important cytokine regulates both cell activation and immune defense (de Martino et al., 2019). The antimicrobial capacity of activated macrophages improves through IFN-γ-induced fusion of phagosomes and lysosomes and autophagy, with the concurrent formation of granulomas. After IFN-γ stimulation, macrophages (and neutrophils) generate reactive oxygen and nitrogen species (ROS/RNS) that restrict mycobacteria, whereas CD8^+^ T cells, NK cells, and Th1 cells contribute to IFN-γ production and cytotoxic effector functions (Cooper et al., 2011). Granulomas organize macrophages and lymphocytes to contain bacilli and limit dissemination, although lesion biology can also create niches that permit pathogen persistence. IFN-γ production sustains a Th1 cell response, which protects against tuberculoid leprosy and stops the bacterial spread (de Sousa et al., 2017).

Mtb and *M. leprae* evade the IFN-γ axis through receptor downregulation, SOCS/IL-10-mediated signaling brakes, type I IFN–driven antagonism, and miRNA-mediated dampening, which can limit responses to IFN-γ–oriented host-directed therapies. Understanding the defense process of IFN-γ enables the development of more effective therapeutic strategies (Fabri et al., 2011).

In this review, we explore IFN-γ signaling in macrophages (phagolysosomal maturation, ROS/RNS, autophagy/LAP), map the evasion mechanisms used by *M. tuberculosis* and *M. leprae*, and appraise preclinical and clinical evidence for IFN-γ–oriented interventions (including TNF-α modulation and GM-CSF adjuvancy), with explicit benefits and risks. We also discuss biomarkers linked to IFN-γ activity (e.g., HIF-1α target gene signatures and functional IFN-γ readouts) to guide patient selection and treatment monitoring.

## 2 Role of IFN-γ in macrophage activation

### 2.1 IFN-γsignaling pathways in macrophages

**Figure 1:**
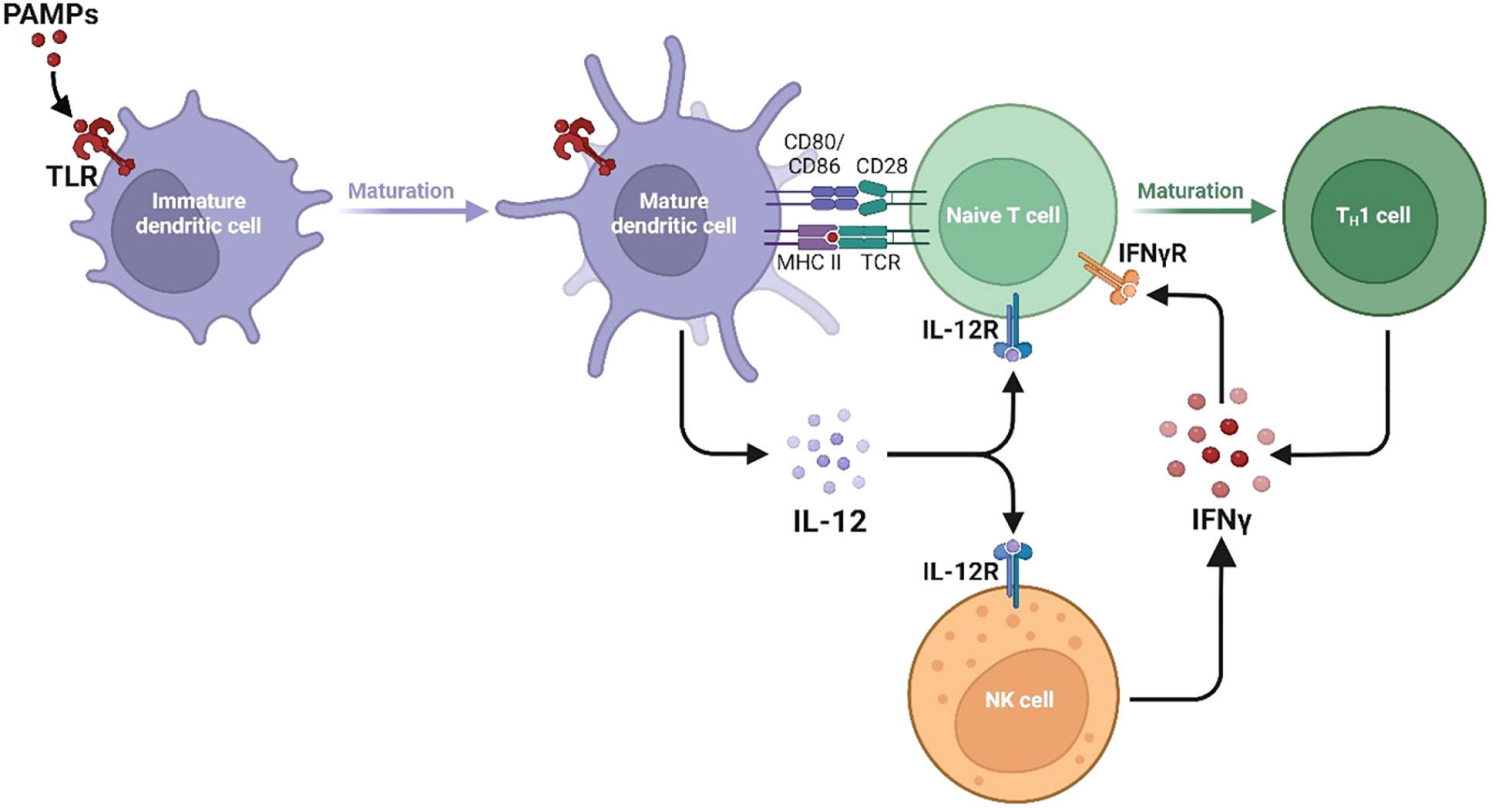
Divergent type I, II, and III interferon signaling pathways and ISG programs. The signalling pathways and gene expression programs activated by Type I, Type II, and Type III interferons are shown, with each type engaging distinct receptor complexes and STAT signalling components. Type I interferons and Type III interferon IFN-γ activate JAK1 and TYK2, leading to phosphorylation of STAT1 and STAT2 and formation of the ISGF3 complex with IRF9. This complex enters the nucleus and binds interferon-stimulated response elements ISREs to induce ISRE-driven genes including MX1, MX2, OAS1, OAS2, OAS3, IFIT1, IFIT2, IFIT3, RSAD2 Viperin, ISG15, EIF2AK2 PKR, IRF7, IRF9, USP18, IFI44, IFI44L, IFI6, IFITM1, IFITM2, and IFITM3. Type II interferon IFN-γ activates JAK1 and JAK2, promoting STAT1 homodimerization and nuclear translocation where it binds gamma-activated sequence GAS elements to drive expression of GAS-regulated genes including NOS2, CYBB, CYBA, NCF1, NCF2, NCF4, RAC2, SOD2, CAT, GPX1, CIITA, HLA-DRA, HLA-DRB1, TAP1, TAP2, PSMB8, PSMB9, GBP1, GBP2, GBP5, IRGM, LAMP1, LAMP2, SQSTM1 p62, IRF1, CXCL9, CXCL10, CXCL11, ACOD1 IRG1, and IDO1. These pathways highlight the specific and overlapping transcriptional programs induced by different interferons to coordinate antiviral immunity, inflammation, antigen processing, and oxidative stress responses.

Through macrophage activation, IFN-γ enables antimicrobial and immunomodulatory functions that fight intracellular pathogens, such as Mtb and *M. leprae* (Bo et al., 2023). The JAK-STAT pathway is activated when IFN-γ binds to the heterodimer IFN-γR, which consists of IFN-γR1 and IFN-γR2 (Liu et al., 2008). STAT1 is phosphorylated, forming dimers that travel into the nucleus to bind GAS sequences, thereby activating the transcription of antimicrobial genes (Speil et al., 2011). The gene-encoded molecules iNOS and ROS have direct pathogen-attack functions in intracellular environments. The degradation of pathogens through phagosome-lysosome fusion is enhanced by IFN-γ, which simultaneously increases MHC expression levels to boost T cell responses (Martin et al., 2022). Chemokines produced by IFN-γ-activated macrophages recruit more immune cells to reinforce the protective defense mechanisms. Mycobacterial pathogens avoid immune detection by secreting IL-10, shutting down STAT1 phosphorylation, and inhibiting macrophage activation. The immune system depends on IFN-γ for protection against mycobacterial infections, even though the bacteria use evasive strategies to bypass the immune response (Tolomeo et al., 2022) (**Figure 1**).

### 2.2 Mechanisms of macrophage activation by IFN-γ

Multiple mechanisms of IFN-γ activation of macrophages enhance their antimicrobial properties, thereby permitting these cells to eliminate intracellular pathogens such as Mtb and *M. leprae.* The key antimicrobial mechanisms include ROS/RNS generation, phagolysosomal maturation, autophagy/LAP, and enhanced antigen presentation. iNOS/NOS2 expression by macrophages upon activation by IFN-γ produces NO, a potent antimicrobial molecule. Controlling mycobacterial infections is critical because they disrupt bacterial cell structures and metabolic functions (Warner, 2014). In addition, IFN-γ upregulates enzymes that increase ROS production, further potentiating the microbicidal activity of macrophages (Tan et al., 2016).

Following IFN-γ engagement of IFN-γR and activation of JAK2–STAT1 (with IRF1), macrophages upregulate oxidant-generating and support enzymes that drive antantimycobacterialemistry. NOS2 (iNOS) is transcriptionally induced to convert L-arginine to NO, and sustained NO flux depends on argininosuccinate synthase-1 (ASS1) to recycle arginine (Rath et al., 2014). In parallel, IFN-γ primes the phagocyte NADPH oxidase (NOX2) by increasing expression/assembly of CYBB/gp91phox and CYBA/p22phox with cytosolic subunits NCF1/p47phox, NCF2/p67phox, NCF4/p40phox, and RAC2, boosting superoxide (O_2_•^−^) generation in phagosomes (Satoh et al., 2016). NO and O_2_•^−^ react to form peroxynitrite (ONOO^−^), which nitrates and oxidatively damages mycobacterial proteins, lipids, and DNA. This oxidative milieu supports autophagy and LAP-linked killing. SOD2 (mitochondrial) and SOD1 (cytosolic) convert O_2_•^−^ to H_2_O_2_, whereas catalase (CAT) and glutathione peroxidases (GPX1/GPX4) detoxify H_2_O_2_ and lipid peroxides to limit host damage while maintaining an antimicrobial redox environment (Dewan et al., 2024). Collectively, IFN-γ heightens NO, O_2_•^−^, and ONOO^−^-mediated killing while preserving macrophage viability, thereby strengthening the control of *M. tuberculosis* and *M. leprae*.

Additionally, IFN-γ promotes macrophage phagosome-lyosomal fusion (Jutras et al., 2008). However, the degradation of engulfed pathogens depends on this fusion process, allowing lysosomal enzymes and acidic conditions within the phagolysosome to break down mycobacterial cell walls and other components. Mtb and *M. leprae* often subvert this pathway; however, IFN-γ counters these evasive mechanisms and restores phagolysosomal killing (Zhai et al., 2019). Another major mechanism is autophagy and LC3-associated phagocytosis (LAP), which cooperate with ROS/RNS to promote the intracellular killing of pathogens. Beyond oxidative killing, IFN-γ drives canonical autophagy and LC3-associated phagocytosis (LAP) in macrophages (Boonhok et al., 2016). Through JAK2–STAT1/IRF1, IFN-γ increases expression/activation of core autophagy machinery (BECN1/Beclin-1, ATG5, ATG7, the ATG12–ATG5–ATG16L1 complex) and selective-autophagy adaptors (p62/SQSTM1, NDP52), promoting LC3-II lipidation and LC3 recruitment to mycobacterial phagosomes (Li et al., 2012). The LAP arm is NOX2-dependent and leverages phagosomal ROS (already primed by IFN-γ) along with Rubicon to decorate phagosomes with LC3, accelerating phagosome–lysosome fusion and hydrolase delivery (Fujiwara et al., 2013). IFN-γ also cooperates with guanylate-binding proteins (GBPs) to enhance the ubiquitin tagging of bacilli-compromised phagosomes, improving receptor-mediated capture (p62/NDP52) and flux. Functionally, IFN-γ–induced autophagy/LAP reduces intracellular CFU and augments MHC-II antigen presentation, acting with ROS/RNS to restrict Mtb and *M. leprae* (Wallet et al., 2017; Mehta and Singh, 2019). Additionally, IFN-γ signaling stimulates the surface expression of MHC, particularly MHC class II, in macrophages (Wijdeven et al., 2018). Collectively, ROS/RNS production, phagosome–lysosome fusion, autophagy/LAP, and enhanced antigen presentation in IFN-γ-activated macrophages are central to host defense against Mtb and *M. leprae* (Dheda et al., 2010).

## 3 Host defense mechanisms mediated by IFN-γ

IFN-γ enhances intracellular killing by licensing macrophages for antimicrobial effector functions, including iNOS-dependent nitric oxide generation and ROS/RNS-linked bactericidal activity, while promoting phagolysosomal maturation and autophagy. These programs cooperate with enhanced antigen presentation to sustain Th1 immunity and cytotoxic T-cell responses, which restrict bacillary replication and support granuloma integrity (Thakur et al., 2019). The benefits of IFN-γ are context-dependent. At high bacillary loads or with virulent Mtb strains, IFN-γ-driven programs can be subverted: apoptosis of infected macrophages may be inhibited, necrotic death favored, and tissue damage amplified, facilitating the spread of bacilli (Nathan and Hibbs, 1991). Moreover, persistent IFN-γ signaling within inflamed lesions can intensify immunopathology when counter-regulatory pathways are disabled or evaded by mycobacteria (Dorhoi et al., 2014). Pathogens utilize mechanisms to disrupt IFN-γ signaling by diminishing the expression of IFN-γ receptors, obstructing the phosphorylation of STAT1, or modifying post-receptor signaling pathways, such as the miR-132/miR-26a–mediated suppression of p300, thereby severing the connection between IFN-γ and its antimicrobial functions (Liu et al., 2008; Ni, 2014). Host factors introduce further heterogeneity; immune status (e.g., HIV co-infection, malnutrition), inflammatory set points, and genetic variation in cytokine/TLR pathways modulate the magnitude and quality of IFN-γ responses, influencing whether the outcomes are protective or pathogenic (González-Torres et al., 2013).

In certain contexts, it can antagonize protective Th1 programs, whereas in others, it restrains permissive macrophage phenotypes in the absence of IFN-γ, underscoring the pathway crosstalk that shapes disease trajectories (Moreira-Teixeira et al., 2016). Recognizing whether IFN-γ is beneficial or harmful has translational value. In drug-resistant TB, adjunctive rhIFN-γ can restore defective macrophage function in selected patients; however, heterogeneity competence and immune evasion limits responses (Khan et al., 2016). Stratifying patients based on bacterial burden, immune status, and IFN-γ pathway integrity may optimize immunotherapeutic use and reduce the risk of immunopathology.

## 4 Therapeutic and diagnostic implications

### 4.1 Potential of IFN-γ as a therapeutic target in tuberculosis

The immune response to Mtb is initiated via pattern recognition receptors, such as Toll-like receptors (TLRs), which recognize bacterial molecules and activate immune cells to produce cytokines and antimicrobial mediators. This response aids in controlling bacterial growth and supports adaptive immunity. Key cytokines, such as IL-12, play a pivotal role in inducing Th1 cells and subsequent IFN-γ production, fully activating macrophages to produce NOS2, which combats Mtb infection. The absence of IFN-γ or NOS2 makes the host highly susceptible to infection, underscoring the importance of these mediators in the immune defense against TB. However, not all immune responses are beneficial. Certain Mtb strains activate TLR4, which, while promoting the expression of protective factors, can also lead to excessive type I IFNs (Mundra et al., 2023). In mice, this exacerbates TB and correlates with severe cases in humans by limiting pro-inflammatory responses, weakening Th1 cell function, and worsening Mtb infection. Teixeira et al. showed that type-I IFN can be protective in the absence of IFN-γ by preventing the transition to a permissive macrophage phenotype and Th2 cytokines, which suppress arginase 1 and increase the production of nitric oxide. This antigenic property of type-I IFN devoid of IFN-γ proposes a multifaceted role in the pathogenesis of TB, as well as possible alternative modes of therapeutic intervention (Moreira-Teixeira et al., 2016).

Mtb-specific CD4 and CD8 T cells develop cytolytic activity in infected macrophages in patients with active TB. Studies on Mtb have indicated that mice infected with Mtb have depleted CD4 T cells, which consequently contributes to widespread infection; however, their presence restricts bacterial proliferation. The drop in CD4 T cell counts witnessed in HIV-1 patients in human beings increases the chances of contracting TB (Okoye and Picker, 2013). In addition, human and murine Mtb-specific CD4 T cells secrete IFN-γ, which plays a significant role in stimulating macrophages and inhibiting intracellular Mtb (Cavalcanti et al., 2012). In contrast, mutations that block the action of IFN-γ result in serious cases of TB and mycobacteremia, which is why IFN-γ plays a vital role in protecting the body against TB. Almeida et al. confirmed that IFN-γ and peripheral blood lymphocytes (PBLs) are also necessary to activate anti-Mtb expression in human macrophages through the participation of IL-12 and inhibition of IL-10, which indicates that IFN-γ may stimulate the bactericidal ability of macrophages (Bonecini-Almeida et al., 1998).

A critical factor in the IFN-γ effect on TB immunity is that it induces TNF-α-dependent macrophage apoptosis. Highly virulent strains of Mtb often disrupt this process, preventing apoptosis and allowing the bacteria to multiply until a certain point is reached, resulting in necrosis and subsequent release of bacilli, leading to further infections. Lee et al. investigated the impact of IFN-γ on macrophages with different bacterial loads. IFN-γ halts bacterial growth at low loads and increases cell death at high loads. This shows the duality of in TB immunity in the context of controlled infection, but possibly destructive in high-burden environments, and the necessity of therapeutic use based on context (Lee and Kornfeld, 2010). Other cytokines, including IL-17 and IL-1b, play significant roles in TB immunity. IL-12 facilitates inflammatory and granuloma stability, whereas IL-17 recruits neutrophils and mucosa. Their response to IFN-γ affects general immunity and disease outcomes. Their inattention to these functions can simplify the immunopathology of TB (Torrado and Cooper, 2010). Optimism has been placed on the role of IL-12 in immunity associated with IFN-γ in studies on IFN-γ-deficient models, where IL-12 helps activate macrophages through endogenous IFN-γ production (Novelli and Casanova, 2004).

Despite the immune-protective role of IFN-γ, Mtb has developed immune evasion mechanisms to diminish its effects. It has been demonstrated that Mtb suppresses IFN-γR expression on macrophages and PBMCs via Tsignalinging, calcium, and kinase pathways, and thus inhibits the effect of IFN-γR on macrophages (Kak et al., 2020). Such an immunosuppressive approach makes the treatment of TB more difficult because high levels of IFN-γ in patients with active TB do not always correlate with sufficient clearance of bacteria. The development of mycobacterial infections is determined by the relationship between pathogens and the host immune system (Chandra et al., 2022). The first contact with mycobacteria triggers the innate immune system, which works in collaboration with the adaptive immune system to control the bacterial proliferation. Recombinant human IFN-γ (rhIFN-γ) has also been found to be promising in MDR-TB as a response to improve macrophage activity in patients with MDR-TB. Khan et al. observed that pre-treatment with rhIFN-γ enhanced the immune response in MDR-TB patients because it restored impaired macrophage functionality (Khan et al., 2016). This result highlights the possibility of using IFN-γ as an adjuvant therapy for treating drug-resistant TB; however, further research is necessary to determine the underlying immunological mechanisms of this action (Palomino and Martin, 2014).

Hirsch et al. showed enhanced IFN-γ and apoptosis in active TB lesions in human tissue studies at disease sites, supporting the local effector role of IFN-γ in bacterial control (Hirsch et al., 2001). Mechanistic murine studies by Lee et al. showed that IFN-γ modulates macrophage fate depending on the bacterial burden containing bacilli at low levels but triggers necrosis at high loads, a duality with therapeutic implications (Lee and Kornfeld, 2010). In clinical settings, aerosolized rhIFN-γ adjunct therapy has improved inflammatory markers and clinical outcomes in patients with pulmonary TB (Gao et al., 2011), and a randomized trial of drug-resistant TB reported faster culture conversion and better outcomes with IFN-γ add-on therapy (Suárez-Méndez et al., 2004). However, these results have been heterogeneous across studies, and systemic side effects such as fever, malaise, and local injection reactions have been reported in some studies. This highlights the need for optimized delivery methods, tailored dosing, and biomarker-guided patient selection strategies.

Research on miRNA involvement has revealed another layer of IFN-γ modulation (Yee et al., 2017). Ni et al. discovered that Mtb infection alters macrophage miRNA expression, particularly upregulating miR-132 and miR-26a, which inhibit p300, a critical IFsignalingling component (Ni et al., 2014). Inhibiting these miRNAs restored IFN-γ responsiveness, suggesting a novel strategy for reversing immune suppression in patients with TB (Wang et al., 2022). IFN-γ enhances autophagic flux, promoting pathogen clearance and restoring defective microbicidal responses in macrophages (Bento et al., 2015). While it may accelerate sputum and culture conversion in difficult TB cases, variability across trials and the potential for IFN-γ to cause tissue damage at high bacterial loads argue for careful dosing, inhaled administration, and guided patient selection using molecular biomarkers.

### 4.2 Biomarkers and diagnostics in tuberculosis

Mtb is phagocytosed by macrophages and resides within phagosomes, evading the immune defenses (Bussi and Gutierrez, 2019). Infected macrophages stimulate cell-mediated immunity by presenting antigens to T cells via major histocompatibility complex (MHC) molecules, leading to T cell activation. Once activated, T cells secrete IFN-γ, which significantly enhances macrophage activity and boosts the expression of MHC class II and co-stimulatory molecules on their surfaces (Sun et al., 2023). This cascade, initiated by IFN-γ binding to IFN-γRα and IFN-γRβ, activates the JAK-signalinglling pathway. This activation triggers the phosphorylation of JAK1 and JAK2, facilitating STAT1 dimerization and nuclear translocation, which promotes the transcription of IFN-γ-inducible genes, such as CIITA and IRF-1, which are crucial for mounting a robust immune response (Kak et al., 2018). However, virulent mycobacteria employ mechanisms to resist immune activation and persist in macrophages (Pieters, 2008). Infected macrophages often exhibit decreased MHC class II expression, which diminishes antigen presentation and impairs IFN-γ response, allowing Mtb to survive within host cells. Hussain et al. revealed that *M. avium* suppresses macrophage activation by downregulating IFN-γR expression and inhibiting the JAK-signalinglling pathway. This suppression limits the expression of IFN-γ-inducible genes and the phosphorylation of STAT1, JAK2, and JAK1, demonstrating a time-dependent mechanism that contributes to Mtb persistence in the host cells (Hussain et al., 1999). The roles of TNF and IFN-γ in the macrophage response to Mtb have also been previously explored. Keane et al. examined murine peritoneal macrophages and found that TNF-α dependent apoptosis played a significant role in bacterial control, whereas IFN-γ pre-treatment killed bacilli independently of apoptosis. This finding underscores the potential strain-specific variability in macrophage responses to Mtb infection, suggesting that the protective effects of IFN-γ may vary depending on the bacterial strain (Keane et al., 2002).

IFN-γ activation drives an HIF-1α–dependent metabolic shift (aerobic glycolysis) in macrophages, thereby supporting antimycobacterial functions. Myeloid HIF-1α deficiency increases TB susceptibility *in vivo*, indicating that HIF-1α activity tracks the effectiveness of IFN-γ-mediated immunity. Braverman et al. demonstrated that IFN-γ activation in macrophages enhances HIF-1α, which regulates essential immune effectors and induces a metabolic shift to aerobic glycolysis, a process crucial for controlling Mtb. Mice lacking HIF-1α in myeloid cells show increased susceptibility to TB, underscoring HIF-1α’s pivotal role in IFN-γ-driven immunity. IFN-γ–activated macrophages require HIF-1α to mount effective antimycobacterial responses; myeloid HIF-1α deficiency increases TB susceptibility *in vivo*, and IFN-γ drives a HIF-1α-dependent shift to aerobic glycolysis, which supports effector functions (Braverman et al., 2016). Practically, because HIF-1α protein is transient, its activity can be tracked via a target-gene signature (e.g., SLC2A1/GLUT1, HK2, PFKFB3, LDHA, PDK1, IRG1/ACOD1) measured in blood or induced-sputum macrophages and by metabolic readouts (e.g., extracellular lactate). The rationale for use during effective therapy is that bacillary load and inflammatory drive fall, and HIF-1α–dependent transcription is expected to normalize, whereas persistent elevation may signal inadequate bacterial control or ongoing lesion activity. Caveats: Hypoxia induces HIF-1α independent of IFN-γ, and site heterogeneity (lung vs. blood) can blur correlations; thus, HIF-1α readouts should be interpreted alongside bacteriological endpoints and complementary immune markers (e.g., TNF/IL-1 modules, IGRA). At the system level, IFN-γ–driven metabolic reprogramming of macrophages requires HIF-1α; myeloid HIF-1α deficiency worsens TB in mice, nominating HIF-1α–regulated transcripts as treatment response biomarkers (Braverman et al., 2016).

**Figure 2:**
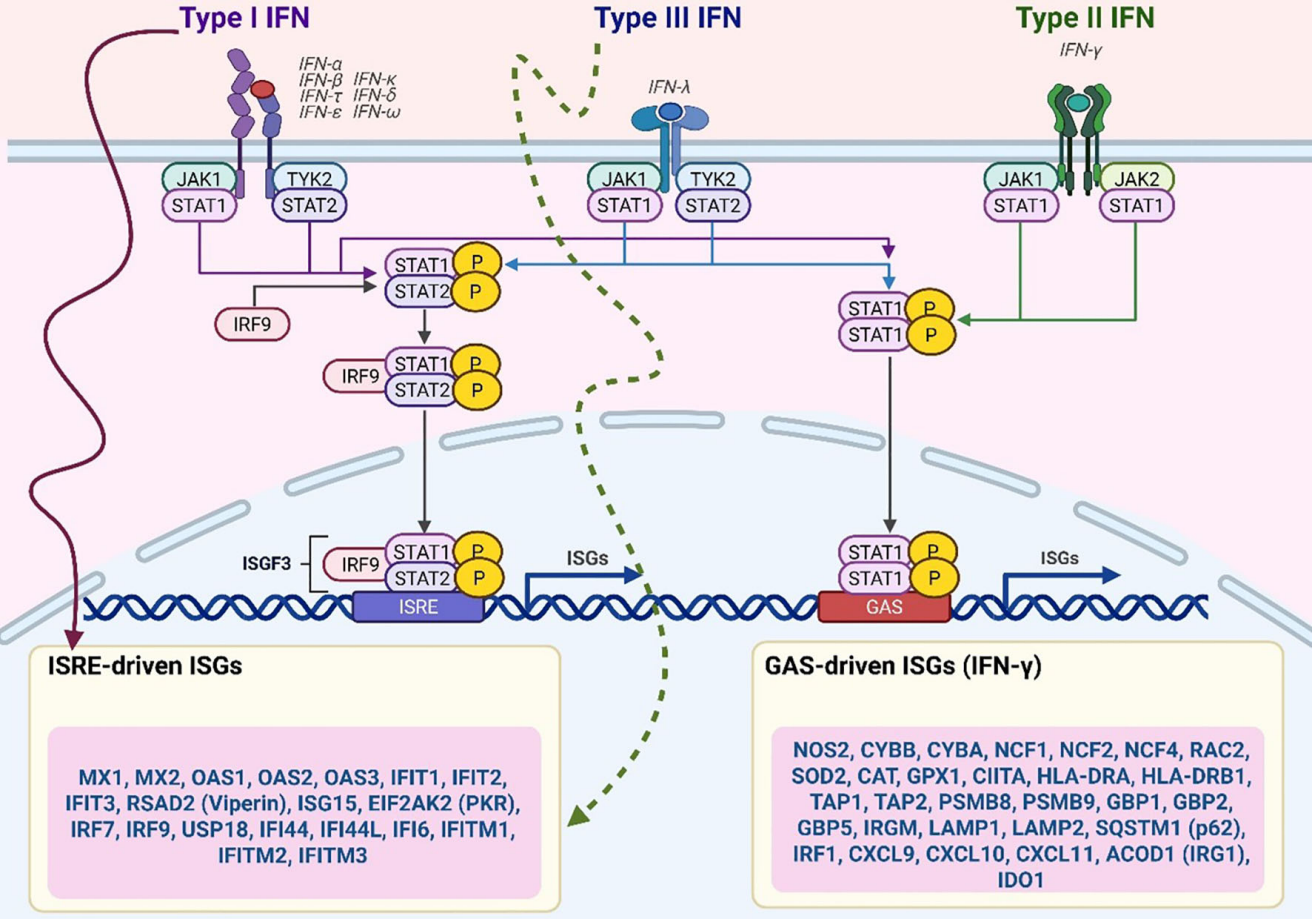
CD4^+^ T cell–driven macrophage activation via IFN-γ and GM-CSF. CD4 T cells activate macrophage antimicrobial responses through cytokines such as IFN-γ and GM-CSF, which promote macrophage polarization and transcriptional reprogramming. IFN-γ signalling enhances the expression of HIF-1α and glycolytic gene programs, whereas GM-CSF signalling through STAT5b synergizes with HIF-1α to drive the expression of pro-inflammatory M1 macrophage transcripts. These M1-related gene programs include glycolytic enzymes and other antimicrobial effectors that restrict Mycobacterium tuberculosis replication. Upstream signals from CD4 T cells are supported by co-stimulatory molecules and inflammatory cues, including TNF α, Type I interferons, CD40, and CD153. Both IFN-γ and GM-CSF signalling promote the accumulation of lipid droplets in macrophages, which are linked to inflammatory functions and potentially fuel antimicrobial activity.

Clinically, interferon-gamma release assays (IGRAs) quantify antigen-specific IFN-γ as functional readouts of T cell priming (Katial et al., 2001), while ESAT-6/CFP-10-based platforms refine TB specificity and are being re-evaluated for immunopathological readouts (Passos et al., 2024). IFN-γ’s role in TB extends beyond direct pathogen control; it interacts with various immune pathways, such as HIF-1α modulation and TNF-associated apoptosis, to influence macrophage function. However, the immune evasion tactics employed by Mtb, such as downregulating IFN-γR and manipulating cytokine production, highlight the need for biomarkers that can signal these disruptions. The identified factors, such as pleural IFN-γ levels, HIF-1α, and IL-1β modulation, provide promising diagnostic insights for assessing TB immune status and treatment response (**Figure 2**).

### 4.3 Potential of IFN-γ as a therapeutic target in leprosy

#### 4.3.1 IFN-γ augmentation in managing leprosy reactions

According to the Ridley–Jopling classification, leprosy is grouped into *tuberculoid (TT), borderline tuberculoid (BT), mid-borderline (BB), borderline lepromatous (BL), and lepromatous leprosy (LL)* forms. A strong cellular immune response to tetanus toxoid (TT) and Bacillus Calmette-Guérin (BCG), often referred to as BT, limits infection, resulting in fewer lesions and lower bacterial loads. In contrast, *lepromatous leprosy* (LL) and *Borderline Lepromatous* (BL) leprosy are linked to the rapid development of bacteria and dysregulated immune reactions, causing enormous infections (Sugawara-Mikami et al., 2022). *M. leprae* persists in skin macrophages and Schwann cells, and the lesion cytokine pattern shows this branch (Lisak et al., 1997). TB lesions have an elevated expression of pro-inflammatory cytokines, such as IFN-γ and TNF-α, which stimulate macrophages to activate and eliminate bacteria. Conversely, lepromatous lesions contain IL-10 inhibits macrophages activity and allows bacterial survival. In LL lesions, *M. leprae* induces foamy macrophages known as Lepra (Virchow) cells a histopathological feature of LL (Mattos et al., 2014). Silva et al. established that LL lesion macrophages release higher levels of IGF-I, inhibiting the JAK/STAT1 cascade and IFN-γ reaction through SOCS3 expression. This is an example of a signaling pathway that helps bacteria survive, and IGF-I suppression has been shown to reenact the macrophage antimicrobial response; hence, IGF-I can be used as a therapeutic choice to stimulate immunity in *LL* (Batista-Silva et al., 2016). Macrophages in TT mainly perform M1 mode of activation, but in *LL* mice, the activation of macrophages is in an M2 alternative state (Batista-Silva et al., 2016). In TT, macrophages predominantly exhibit an M1 activation phenotype, whereas in *LL-infected* mice, they adopt an M2 alternative activation state (Strizova et al., 2023). Studies suggest that BCG vaccination, which is protective against leprosy, may promote an M1 macrophage response. Fallows et al. demonstrated that *M. leprae* exposure inhibits M1 polarization, likely through its phenolic glycolipid (PGL-1). Notably, monocytes from BCG-vaccinated infants produce higher levels of TNF-α and IL-1β in response to *M. leprae*, suggesting that BCG vaccination may counteract the immunosuppressive effects of *M. leprae* by promoting a protective M1 macrophage response (Fallows et al., 2016).

Macrophage heterogeneity in leprosy reflects the cytokine environment, particularly with two distinct macrophage subsets: GM-CSF-mediated macrophages (GM-Mφ) and M-CSF-mediated macrophages (M-Mφ). Both subsets are equally vulnerable to infection *in vitro*, although M-Mφ *in vitro M. leprae*-infected M-Mφ do not stimulate CD4+ T cells, despite stimulation with CD40 ligand and IFN-γ. M-Mφ generate a large amount of IL-10 in response to *M. leprae*, which is associated with latent infection and disease progression (Silva et al., 2024). The use of modified strains of BCG, such as recombinant BCG-SM, can augment immunity against *M. leprae* as it fully activates T cells. Makino et al. built a host-modified *M. bovis* BCG strain, which expresses *M. leprae*-derived MMP-II, stimulating the generation of macrophages and GM-CSF. This change led to the activation of IFN-γ-producing CD4+ T cells more effectively than the control BCG, largely because GM-CSF prevented the production of IL-10 in macrophages. GM-CSF enhances T cell response through macrophages by inhibiting IL-10, which is an encouraging development in the treatment of leprosy in new ways (Makino et al., 2009). The activities of IFN-γ and TNF-α in the production of hsp65-specific cytotoxic T cells (CTLs) during *M. leprae* infection are crucial. Sasiain et al. reported that cytokines, including IL-6, used with IFN-γ or IL-2, stimulate CTL activity in patients with paucibacillary and multibacillary leprosy, affecting both CD4 and CD8 CTLs. In contrast, IL-4 and IL-10 suppress CTL production, which is an example of cytokine action in the immune response to leprosy (Del C Sasiain et al., 2008). In the armadillo model, recombinant armadillo IFN-γ (rDnIFN-γ) activates macrophages and improves the control of intracellular pathogens, providing a species-relevant platform for developing leprosy immunotherapy (Peña et al., 2008). Ex vivo human models have shown that *M. leprae* dampens dendritic cell antigen presentation and co-stimulation, blunting IFN-γ-producing T-cell responses, thereby providing mechanistic support for IFN-γ augmentation strategies (Hashimoto et al., 2002). Type I IFN–driven suppression of the vitamin D antimicrobial pathway in human monocytes can be reversed by blocking IFNAR, restoring CYP27B1 induction, and downstream killing programs (Fabri et al., 2011), whereas GM-CSF-augmented macrophage activation enhances IFN-γ–positive T-cell priming in recombinant BCG systems (Makino et al., 2009). It strengthens the Th1/M1 axis and macrophage bactericidal functions in *LL* and enhances host-directed therapy. The risk of triggering reactions (T1R/ENL) in multibacillary disease; benefits may depend on the cytokine milieu (IL-10/IGF-I high) and require careful clinical selection. Fink et al. demonstrated that cytokine modulation can improve CTL generation against *M. leprae.* IFN-γ, IL-6, and IL-6 combined with IL-2 enhanced CTL development, whereas IL-4 suppressed it. Notably, IFN-γ counteracted IL-4’s inhibitory effect, indicating the potential for cytokine-based modulation to enhance immune responses in leprosy treatment (FINK et al., 1993). IFN-γ is crucial for managing leprosy reactions by promoting macrophage activation and CTL responses and modulating immune suppressive mechanisms. Studies have revealed that manipulating cytokines, such as IFN-γ, TNF-α, and GM-CSF, offers therapeutic advantages, especially in the more severe forms of leprosy. In severe leprosy reactions (ENL), TNF-α modulation shows the strongest clinical signal. A randomized, double-blind, double-dummy trial showed that thalidomide rapidly controlled ENL and reduced steroid requirements (dose-comparison RCT) (Villahermosa et al., 2005). For refractory ENL, small clinical experiences report benefits from anti-TNF biologics, such as infliximab, including an NEJM case and subsequent series; however, pharmacovigilance and systematic reviews note a risk signal for leprosy occurring under anti TNF-α therapy, necessitating careful selection and monitoring (Faber et al., 2006; Cogen et al., 2020; Mendes et al., 2022). On the preclinical/translational side, a recombinant BCG engineered to secrete *M. leprae* major membrane protein-II induced GM-CSF in macrophages and enhanced IFN-γ producing CD4^+^ T-cell priming, supporting GM-CSF–based adjuvancy (Makino et al., 2009). Ex vivo human studies have shown that *M. leprae* impairs dendritic cell antigen presentation/co-stimulation, blunting IFN-γ T cell responses (Hashimoto et al., 2002). Moreover, blocking signaling reactivates the vitamin D antimicrobial pathway and reduces the survival of *M. leprae* in human monocytes, suggesting a way to safely alter the cytokine environment towards IFN-γ–dependent control without causing adverse reactions ​ (Zavala et al., 2018). Thalidomide is known for its rapid effectiveness in controlling ENL and reducing the need for steroid therapy. GM-CSF acts as an adjuvant to enhance antigen presentation and facilitate Th1 cell activation. Additionally, specifically targeting and reducing type I IFN can help restore the cell-killing function linked to IFN-γ production. However, anti-TNF-α biologics are associated with a potential risk of leprosy, and directly increasing IFN-γ levels in cases of multibacillary disease may trigger adverse reactions in patients. Furthermore, the current GM-CSF findings are limited to preclinical studies and require further clinical trials for confirmation of their validity. Targeting specific pathways, such as IGF-I inhibition in *LL* and IL-10 suppression, could potentially enhance immune activation and support bacterial clearance, offering promising avenues for therapeutic intervention.

#### 4.3.2 Targeting IFN-γ pathways to improve immune response in *lepromatous leprosy*

Leprosy is an immunohistopathological spectrum ranging from *LL* with a Th2-type cytokine profile and weak cell-mediated immunity (CMI) to TT, where a strong Th1 response is observed. The adaptive immune response in leprosy, primarily regulated by T cells (including CD4+, CD8+, CD1-restricted, and γδ T cells), shapes disease presentation (Sadhu and Mitra, 2018). Mononuclear phagocytes, mainly macrophages (Mφ), serve as the primary host cells for *M. leprae.* In *LL*, inactivated Mφ permit unchecked bacterial growth, whereas in TT, activated Mφ are efficient in killing *M. leprae* with the aid of IFN-γ. However, in the *LL* phase, immune evasion tactics diminish the effectiveness of macrophages (Montoya et al., 2009). Hagge et al. created a model to examine *M. leprae* granulomas in LL cases, with ACT Mφ playing a significant role in reactive nitrogen intermediates and cell-to-cell interactions that prevent *M. leprae* activity. the significance of macrophage activation states in the control of bacterial viability in *LL* lesions and proposed that the enhancement of macrophage activation by targeting signaling might be used to overcome the immunosuppressive response of *LL* (Hagge et al., 2004). Macrophage involvement in granulomas has also been studied in mycobacterial infections. Wang et al. created granuloma-like aggregates using a co-culture model of human macrophages and PBMCs to investigate *M. leprae* granulomas. M1 (pro-inflammatory) and M2 (anti-inflammatory) macrophages were present, and *M. leprae* was active in the cells. This model provides information on granuloma formation and highlights the complexity of macrophage polarization in granulomas. Such immune transactions may prove useful in realizing the future in disease control measures, particularly where *LL* is also capable of eliminating bacteria through immune modulation (Wang et al., 2013). *LL* has been associated with the immunosuppressive state over one receptor, CD163, which is expressed exclusively on monocytes and macrophages. This receptor mediates the synthesis of anti-inflammatory cytokines, such as IL-10, contributing to the formation of a positive feedback loop that promotes the expression of CD163 (Evans et al., 2013).

**Figure 3:**
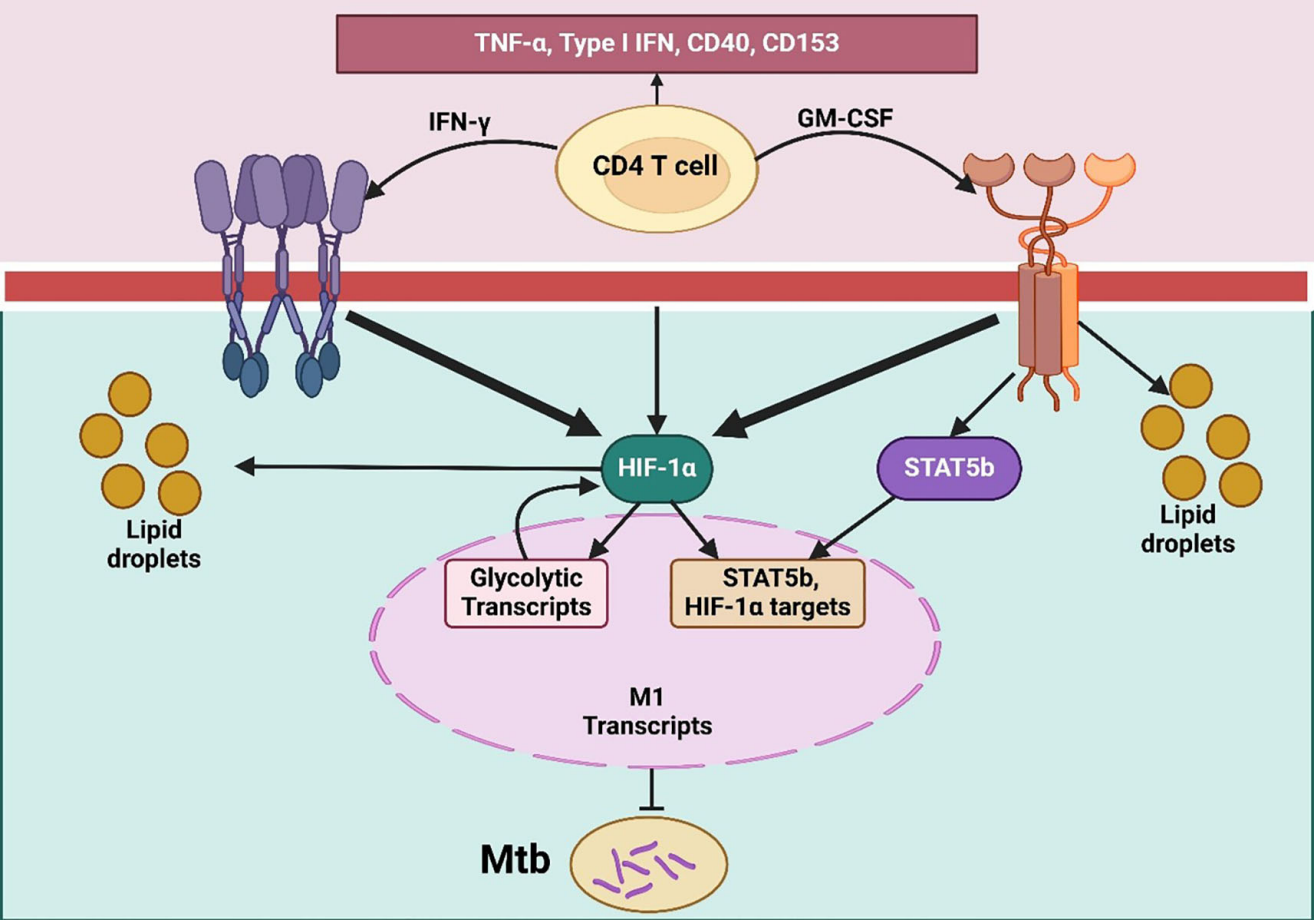
TLR-primed DC–T/NK cell axis orchestrating IL-12–dependent Th1 differentiation. It illustrates the T cell activation and differentiation processes through dendritic cells, naïve T cells, and NK cells in response to pathogen-associated molecular patterns (PAMPs). PAMPs bind to toll-like receptors (TLRs) on immature dendritic cells, prompting their maturation. Mature dendritic cells then present antigens to naïve T cells using MHC II, CD80, and CD86 molecules alongside the secretion of IL-12. IL-12 binds to its receptor (IL-12R) on naïve T cells and NK cells, activating both cell types. Activated NK cells release IFN-γ, which enhances the maturation of naïve T cells into Th1 cells. This differentiation into Th1 cells is crucial for a strong cell-mediated immune response, facilitated through both direct and indirect T-cell activation by TLR agonists.

Moreover, the vitamin D pathway overlaps with IFN-γ-mediated responses in patients with leprosy. However, although vitamin D is utilized in the generation of antimicrobial responses by the immune system, type I IFN is utilized by *M. leprae* to inhibit the vitamin D-dependent antimicrobial response of IFN-γ (Sassi et al., 2018). Zavala et al. also showed that *M. leprae* suppresses the macrophage response to vitamin D by establishing type-I IFN. Type-I IFN receptors, which are required to stimulate the vitamin D pathway, were blocked, and monocyte activation and CYP27B1 expression were recovered by blocking these receptors. The present study hypothesized that with the assistance of IFN-γ regulation, the immune system against *LL* could be bolstered by restoring the functions of the vitamin D pathway (Zavala et al., 2018). Leprosy susceptibility and immune responses are also influenced by genetic factors. Differences in leprosy outcomes are linked to polymorphisms in Th1-related genes such as TNF-α, IL-12, and TLR. Variants of TLR1 have been linked to altered monocyte reactions and protection against reversal reactions, whereas TLR2 and TLR4 variants are linked to overall vulnerability to leprosy (Santana et al., 2017). According to Sinsimer et al., *M. leprae* represses monocyte pro-inflammatory reactions by triggering negative regulators, including MCP-1 and IL-1Ra, and by diminishing the production of IL-6 through signaling pathway. This study highlights the ability of *M. leprae* to inhibit NF-κB and caspase-1 activation, which restricts cytokine release and dampens immune responses. Notably, this suppression primes cells for heightened TNF-α and IL-10 production upon secondary stimulation, demonstrating *M. leprae*’s active modulation of host immunity to persist in the host. *LL* presents unique challenges owing to immune suppression mechanisms that limit IFN-γ and related pathways. Targeting specific elements, such as enhancing macrophage activation through vitamin D pathways, blocking immunosuppressive receptors such as CD163, and understanding genetic factors that influence host immunity, could offer new avenues for therapeutic interventions. By strengthening IFN-γ responses and overcoming *M. leprae* immune evasion strategies, future treatments may shift the immune response from an *LL* phenotype toward a more effective bactericidal state (**Figure 3**).

### 4.4 Biomarkers and diagnostics in leprosy

*M. leprae* and other intracellular pathogens regulate macrophage metabolism to prevent host defense. Nevertheless, macrophages can be activated via innate and adaptive immune responses to initiate antimicrobial processes (Bowdish et al., 2007) signaling occurs in response to the identification of bacterial lipoproteins through TLR2/1 and amplifies the effects of macrophages, resulting in the synthesis of vitamin D-dependent antimicrobial peptides by IFN-γ. The immune system plays a major role in the evolution of clinical manifestations of leprosy. *TT* is linked to suppressed bacterial growth and small lesions, whereas LL is linked to extensive infections and a large bacterial load (Fonseca et al., 2017). A reversal reaction represents an immune shift wherein LL lesions move toward a TT phenotype, reducing the number of bacteria and leading to improved clinical outcomes (Luo et al., 2021). In leprosy, S100A12 has evolved to become an essential antimicrobial agent in macrophages. Realegeno et al. found that S100A12, which is induced by TLR2/1, is directly lethal to mycobacteria and is more highly expressed by TT than by LL. This suggests that S100A12 can be used as a marker of successful immune control of *M. leprae* and can be implemented as a marker of disease progression or dietary compliance (Realegeno et al., 2016).

The action of IFN-γ against *M. leprae* aims to enhance antimicrobial activity by stimulating macrophages to enhance antigen processing (Massa et al., 2023). However, these pathways are complicated by species differences, which affect their effectiveness. As a rule, IFN-γ in rodents induces the generation of reactive nitrogen intermediates (RNI) that help destroy mycobacterial pathogens (Firmani and Riley, 2002). Human IFN-γ directed macrophages also generate insufficient nitric oxide, which limits their use in destroying Mycobacteria (Qualls et al., 2012). One of the most important model organisms in leprosy research, the armadillo (Dasypus novemcinctus), shares IFN-γ responses with humans (Sharma et al., 2013). Tambunan et al. demonstrated that rDnIFN-γ stimulated macrophages to control intracellular pathogens, such as Toxoplasma gondii. Unlike rodents and armadillos, however, IFN-γ-stimulated human macrophages do not produce nitrite and do not prevent *M. leprae* infection, which highlights the relevance of the armadillo model in studying human-like responses to leprosy. T cells (CD4+ and CD8+) play an important role in mycobacterial infections because they produce IFN-γ that activates macrophages (Tambunan et al., 2018). CD8+ T cells have recently been recognized for their ability to secrete IFN-γ and directly kill mycobacteria by producing perforin and granulysin (Woodworth et al., 2008). CD1+ CD83+ monocyte-derived DCs in leprosy lesions contribute to protective immunity by stimulating T cells (Sieling et al., 1999). However, Hashimoto et al. found that *M. leprae*-infected DCs displayed reduced HLA and CD86 expression, limiting their capacity to activate IFN-γ-producing T cells compared to the response induced by *M. bovis* BCG. Masking the *M. leprae* PGL-1 antigen improved T cell activation, suggesting that *M. leprae* evades DC-mediated immunity, which may impair host defense during the early stages of infection. Various diagnostic tests have been developed for leprosy, although with mixed success (Lopes-Luz et al., 2023). Serological detection of PGL-I, an *M. leprae-specific* antigen, effectively identifies antibodies in patients with multibacillary leprosy but is less effective in those with paucibacillary leprosy (Richardus et al., 2017). Tests based on cell-mediated immunity, such as the IFN-γ whole blood assay and delayed-type hypersensitivity skin test, have also shown potential (Katial et al., 2001; Wu et al., 2025). Advances in comparative mycobacterial genomics may improve diagnostic specificity. Spencer et al. used genome analysis to identify *M. leprae*-specific genes and tested recombinant proteins and synthetic peptides for IFN-γ responses across patient groups. They observed that certain 9-mer peptides elicited specific IFN-γ responses in leprosy patients, especially activating CD8+ T cells, indicating that these peptides may serve as valuable diagnostic tools for early detection and epidemiological surveys (Spencer et al., 2005) (**Table 1**).

**Table 1:** Comprehensive overview of research studies examining the role of IFN-γ (Interferon-gamma) and related immune pathways in macrophage responses to *Mycobacterium tuberculosis* (Mtb) and *Mycobacterium leprae* (M. leprae) infections.

Study objective	Outcome	Focus	Mechanism	Pathway	Reference
Assessing IFN-γ-independent macrophage response to Mtb	Type I IFN protects against Mtb in the absence of IFN-γ	Type I IFN protects against Mtb	Inhibits alternative macrophage activation	Th2 cytokines suppressed, NO synthase promoted	(Moreira-Teixeira et al., 2016)
Examining IFN-γ and PBL’s role in anti-Mtb activity	Enhanced macrophage bactericidal effects	IFN-γ and PBL activate anti-Mtb effects	Enhances IL-12, reduces IL-10	Nitric oxide synthase in macrophage-killing	(Bonecini-Almeida et al., 1998)
Investigating IFN-γ’s effects on macrophages based on Mtb load	IFN-γ limits bacterial replication at low loads	IFN-γ inhibits bacterial replication based on load	Inhibits replication at low, accelerates death at high	Macrophage response varies with load	(Lee and Kornfeld, 2010)
Understanding Mtb’s impact on IFN-γ receptor expression	Downregulation of IFN-γ receptor on macrophages	Mtb downregulates IFN-γ receptor on macrophages	Suppresses IFN-γR via TLR, PKC	Calcium, ERK-MAPK pathways affected	(Kak et al., 2020)
Exploring IL-12’s enhancement of IFN-γ-driven macrophage activation	Increased TNF-α and NO release	IL-12 enhances TNF-α/NO release via IFN-γ	Induces IFN-γ to boost TNF-α	IFN-γ-deficient mice confirm mechanism	(Xing et al., 2000)
Investigating rhIFN-γ in enhancing immune function in MDR-TB patients	Improved macrophage function with rhIFN-γ treatment	rhIFN-γ enhances macrophage function in MDR-TB	Adjuvant for improved immune response	Boosts macrophage bactericidal action	(Khan et al., 2016)
Examining miRNA’s role in IFN-γ pathway inhibition	Mtb uses miRNAs to limit IFN-γ signaling	Mtb uses miRNAs to inhibit IFN-γ signaling	miR-132 and miR-26a regulate p300	Impairs IFN-γ-mediated immunity	(Ni et al., 2014)
Assessing IL-6’s impact on IFN-γ-induced autophagy	IL-6 inhibits autophagy, which is crucial for bacterial clearance	IL-6 inhibits IFN-γ-induced autophagy	Downregulates Atg12-Atg5 complex	Blocks autophagosome formation	(Dutta et al., 2012)
Investigating cytokine roles in macrophage and DC response	Unique immune roles of macrophages and DCs in Mtb	Macrophages and DC roles in Mtb infection	Macrophages produce IL-10, DCs enhance IL-12	Different cytokine production levels	(Giacomini et al., 2001)
Exploring IFN-γ levels in TB patient samples	Higher IFN-γ in pleural fluid than in plasma	Increased IFN-γ in pleural fluid in TB	Apoptosis observed in T cells	Linked to the immune activation cycle	(Hirsch et al., 2001)
Analyzing TNF/IFN-γ in Mtb response	IFN-γ increases macrophage-killing	TNF/IFN-γ response in murine macrophages	Bacterial control without apoptosis	Varies by murine strain	(Keane et al., 2002)
Examining M. avium’s suppression of IFN-γ receptor	Decreased IFN-γ signaling pathway activity	Mycobacterium avium downregulates IFN-γ pathways	Reduces JAK-STAT pathway signaling	Impacts IFN-γR expression	(Hussain et al., 1999)
Investigating CD4 T cell activation without IFN-γ	GM-CSF supports macrophage control without IFN-γ	CD4 T cells activate Mtb control without IFN-γ	GM-CSF and HIF-1α facilitate macrophage response	IFN-γ-independent immune mechanism	(Van Dis et al., 2022)
Exploring HIF-1α’s role in IFN-γ response	IFN-γ increases HIF-1α for effective Mtb control	HIF-1α mediates IFN-γ infection control	Shifts metabolism to aerobic glycolysis	Essential for infection control	(Braverman et al., 2016)
Evaluating IGF-I’s impact on IFN-γ response in LL	IGF-I reduces IFN-γ signaling in LL lesions	IGF-I inhibits JAK/STAT1 in LL	IGF-I induces SOCS3 to limit the immune response	Promotes Mtb survival in LL	(Batista-Silva et al., 2016)
Investigating BCG vaccination’s impact on monocytes	BCG primes monocytes for pro-inflammatory response	BCG promotes M1 response in leprosy	Influences cytokine production via PGL-1	Counteracts *M.L*’s suppression	(Fallows et al., 2016)
Assessing cytokine role in CTL generation	IFN-γ enhances CTL responses	Cytokine modulation of CTL generation	IL-6 and IL-2 support CTL development	IFN-γ counters IL-4 suppression	(FINK et al., 1993)
Evaluating CTL activity in leprosy	IFN-γ and TNF-α enhance CTL in MB/PB patients	IFN-γ and TNF-α role in CTL activity	IL-6, IFN-γ boost CD4 and CD8 responses	CTL generation for M. leprae	(Del C Sasiain et al., 2008)
Testing modified BCG strain for enhanced immune response	Boosts GM-CSF and IFN-γ-producing T cell activation	BCG strain enhances macrophage activation	Promotes T-cell response, limits IL-10	Supports macrophage-T cell synergy	(Makino et al., 2009)
Examining Mφ effect on M. leprae in granulomas	Activated Mφ reduces M. leprae viability	M. leprae immune modulation in granulomas	Nitrogen intermediates and cell contact	Impacts on LL lesion environment	(Hagge et al., 2004)
Investigating antimicrobial protein role in leprosy	S100A12 essential for killing Mtb and M. leprae	S100A12 in macrophage antimicrobial activity	Activated by TLR2/1L and IFN-γ	Higher in tuberculoid than LL	(Realegeno et al., 2016)
Analyzing transcription factor responses in leprosy	STAT-6 activated in tuberculoid, CREB in LL	T cell transcription modulation in leprosy	Differing cytokine profile by disease type	Links to Th1/Th2 immune response	(Upadhyay et al., 2019)
Evaluating the armadillo model for leprosy studies	rDnIFN-γ activates armadillo macrophages	Armadillo model of IFN-γ in leprosy	Induces macrophage intracellular activity	Lacks nitrite production for M. leprae	(Peña et al., 2008)
Testing DC role in IFN-γ stimulation by M. leprae	M. leprae evades DC immunity with low HLA/CD86 expression	IFN-γ responses reduced in DC by M. leprae	Masked by PGL-1 antigen in M. leprae	Limits T-cell activation early in infection	(Hashimoto et al., 2002)
Identifying diagnostic peptides for leprosy	Specific peptides trigger IFN-γ in leprosy patients	IFN-γ responses for early detection of M. leprae	CD8+ T cells activated by 9-mer peptides	Diagnostic potential for epidemiology	(Spencer et al., 2005)

## 5 Comparative analysis of IFN-γ’s role in Mtb and *M. leprae* infections

Mycobacteria, including Mtb and *M. leprae*, evade the microbicidal functions of macrophages and impair antigen presentation, undermining protective immunity against these pathogens. In leprosy, the clinical manifestations strongly correlate with immune system activation. TT patients exhibit robust T cell responses, whereas lepromatous patients display weakened T cell reactivity and elevated antibody levels. T-cell responses, particularly those producing IFN-γ, are crucial for protection (Geluk, 2018; Wang et al., 2025). Since *M. leprae* is incurable in culture, determining efficient antigens is critical for developing a vaccine against *it*. One of the potent T-cell targets of TT is heat shock proteins (hsps), which refer to ML GroES, and provoke high levels of IgG1 antibodies associated with disease progression (Koliński et al., 2016). This is due to a decrease in IFN-γ levels when IgG1 is high, and the levels of IFN-γ are fundamental to macrophage activation, which makes it difficult to design vaccines (Leopold Wager et al., 2018). Hussain et al. established that ML GroES has the potential to provoke T cell responses in tuberculoid leprosy and healthy contacts, leading to IgG1 antibodies that are linked to severe disease. This underlies the complexity of immune responses to leprosy and implies a looser concept of vaccine pathogenesis and development (Hussain et al., 2004; Wang et al., 2024). Although infection with *M. ulcerans* is not the focus of interest when writing this review, its comparison with *M. leprae* provides a valuable immunological background. The variety of adaptive programs of mycobacteria is highlighted because immunological evasion in *M. ulcerans* and *M. leprae* is highly divergent. *M. ulcerans* causes the expression of the macrolide-toxin mycolactone, which is cytotoxic, macrophage-inhibitory, and IFN-γ is crucial for the extracellular survival of bacilli. These differences are also known, which contributes to the possibility of different types of mycobacterial species to meddle the host immunity using certain molecular tools, thereby determining the further evolution of certain diagnostic and immunotherapeutic procedures. *M. leprae* is an obligatory intracellular microorganism that does not cause any immune response and can survive by multiplying in phagocytic and Schwann cells (Tian et al., 2022). Röltgen et al. argued that they are important in developing special vaccines, diagnostic procedures, and therapies, especially because the initial manifestations of leprosy and Buruli ulcer are often misunderstood (Röltgen et al., 2020).

Management of latent tuberculosis infection (LTBI) can be considered one of the prime steps in high-burden localities where other agencies, including the CDC and WHO, provide the principles of LTBI diagnosis and management, specifically in high-risk groups (Chen et al., 2019). Pai et al. emphasized the necessity of diagnosing LTBI using tests such as the interferon-gamma release assay (IGRA) and the tuberculin skin test (TST). However, these tests cannot distinguish between LTBI and active TB in immunocompromised patients. Further research is needed to identify biomarkers that can adequately predict the development of LTBI into an active disease (Pai and Behr, 2016). Multiparameter flow cytometry has helped in understanding the role of T cell efficiency in mycobacterial infections (Estévez et al., 2020). Protection against TB is correlated with the presence of multifunctional Th1 cells that secrete IFN-γ, IL-2, TNF-α, and other cytokines (Zeng et al., 2018). According to Santos et al., *M. leprae*-specific multifunctional T cells that generate IFN-γ and IL-2 are more common in healthy contacts of leprosy patients and in paucibacillary cases than in multibacillary cases. The implication of this Th1-biased response is that it plays a protective role against *M. leprae*, which might be useful in the future for developing a human-specific leprosy vaccine. TB vaccines have logistical and economic advantages in areas where TB and leprosy may coexist in the future (Duthie et al., 2014). However, the possibility of cross-protection remains to be determined. Such ID83 and ID93 TB vaccine candidates, in combination with TLR4L adjuvants, have demonstrated this (Enriquez et al., 2021; Song et al., 2022). Duthie et al. found that these vaccines induced IFN-γ production in patients with TB and PB leprosy and healthy contacts. At the same time, TB-vaccinated mice showed decreased growth of *M. leprae*, indicating that vaccines might be used to control leprosy (Duthie et al., 2014). RBCG30, a promising antigen 85B of Mtb antigen overexpressing recombinant BCG vaccine, is another approach for dual vaccination (Hoft et al., 2008). Gillis et al. established that rBCG30 was more efficient in providing immunoprotective effects against Mtb and M. bovis and in inducing immunological responses, including the release of more IFN-γ, in a phase I human trial than conventional BCG did. When improved with antigen 85B, rBCG30 was more protective against *M. leprae* in mouse models, suggesting that rBCG30 can be used as a combination vaccine against TB and leprosy in endemic countries (Gillis et al., 2014).

TB is diagnosed in the presence of culture filtrate proteins of Mtb, such as T-ESAT-6, which are recognized by CD4+ Th1 cells (Passos et al., 2024). This antigen is not found in *M. leprae* or nontuberculous mycobacteria (NTM) and serves a good purpose in differentiating between TB and environmental exposure to Mycobacteria. Geluk et al. determined the diagnostic potential of *M. leprae* ESAT-6 (L-ESAT-6), a homologue of TB T-ESAT-6. L-ESAT-6 prompted the production of IFN-7 by individuals with leprosy, signifying the cross-reactivity of L-ESAT-6 and T-ESAT-6, with diagnostic consequences for individuals with high TB and leprosy co-endemicity (Geluk et al., 2002). In contrast to Mtb ESAT-6, which is useful for detecting TB-specific immune responses, *M. leprae* ESAT-6 is a problem in TB and leprosy areas because of its cross-reactivity with other mycobacteria (Zhuang et al., 2024). Geluk et al. defined Mtb CFP-10 and ESAT-6 as valuable diagnostic antigens for TB; however, their diagnostic specificity against the cross-reacting homologs of *M. leprae* is limited because of their cross-reactivity (Geluk et al., 2004).

## 6 Conclusion and future perspectives

In summary, evidence from murine models and human clinical/lesional studies delineates how IFN-γ orchestrates macrophage antimicrobial programs and how Mycobacteria evade this axis. We compared the protective and pathological facets of IFN-γ in tuberculosis and leprosy and appraised its translational potential in drug-resistant TB, alongside the regulatory circuits that tune this response (IL-12/STAT1, miRNA-mediated brakes, autophagy/LAP). The limitations of the current evidence base include small, heterogeneous clinical trials, variability in the dose, route, and timing of IFN-γ or cytokine-modulating interventions, and limited prospective biomarker-anchored studies, particularly in leprosy. These considerations inform our recommendations for biomarker-guided patient selection, optimized delivery, and adequately powered clinical trials in the future. Pathogenic mycobacteria, Mtb and *M. leprae*, enter host cells, but the production of ROS/RNS can be triggered by IFN-γ through phagosome-lysosome fusion and autophagy. Granuloma formation coupled with pathogen confinement plays a crucial role in IFN-γ. Macrophage killing is impaired by the mycobacterial release of signaling (e.g., decreased IFN-γR expression and inhibitory IL-10/SOCS induction), potentially suppressing the impact of IFN-γ adjuncts and slowing the response to treatment. This review evaluates how IFN-γ functions as a powerful antimicrobial defense system and how it is susceptible to microbial regulatory mechanisms. Immune activation requires IFN-γ; however, further research is needed to determine the influence of host and pathogen factors on this process. The scientific applications of IFN-γ pathway maximization offer important outcomes for MDR-TB treatment and the management of severe leprosy. Widespread therapeutic approaches need to optimize the anti-inflammatory properties of IFN-γ while managing any negative side effects at the same time. Future investigations should focus on improving signaling, along with additional therapeutic studies and the development of biomarkers to track treatment responses. New technical innovations will create opportunities to develop medicines that specifically target mycobacterial infections.
